# Modular development of a prototype point of care molecular diagnostic platform for sexually transmitted infections

**DOI:** 10.1016/j.medengphy.2016.04.022

**Published:** 2016-08

**Authors:** Manoharanehru Branavan, Ruth E. Mackay, Pascal Craw, Angel Naveenathayalan, Jeremy C. Ahern, Tulasi Sivanesan, Chris Hudson, Thomas Stead, Jessica Kremer, Neha Garg, Mark Baker, Syed T. Sadiq, Wamadeva Balachandran

**Affiliations:** a College of Engineering, Design and Physical Sciences, Brunel University London, Kingston Lane, Uxbridge, UK; b Oceans and Atmosphere Flagship, Commonwealth Science and Industrial Research Organisation (CSIRO), Hobart, Tasmania 7001, Australia; c Institute for Infection and Immunity, St George's University of London, Cranmer Terrace, London SW17 0RE, UK

**Keywords:** Microfluidics, Microengineering, Point-of-care, Molecular diagnostics

## Abstract

This paper presents the design of a modular point of care test platform that integrates a proprietary sample collection device directly with a microfluidic cartridge. Cell lysis, within the cartridge, is conducted using a chemical method and nucleic acid purification is done on an activated cellulose membrane. The microfluidic device incorporates passive mixing of the lysis-binding buffers and sample using a serpentine channel. Results have shown extraction efficiencies for this new membrane of 69% and 57% compared to the commercial Qiagen extraction method of 85% and 59.4% for 0.1 ng/µL and 100 ng/µL salmon sperm DNA respectively spiked in phosphate buffered solution. Extraction experiments using the serpentine passive mixer cartridges incorporating lysis and nucleic acid purification showed extraction efficiency around 80% of the commercial Qiagen kit. Isothermal amplification was conducted using thermophillic helicase dependant amplification and recombinase polymerase amplification. A low cost benchtop real-time isothermal amplification platform has been developed capable of running six amplifications simultaneously. Results show that the platform is capable of detecting 1.32×10^6^ of sample DNA through thermophillic helicase dependant amplification and 1×10^5^ copy numbers *Chlamydia trachomatis* genomic DNA within 10 min through recombinase polymerase nucleic acid amplification tests.

## Highlights

• A low cost, optical, POC molecular diagnostic platform.
• Sample preparation using a paper membrane.
• Isothermal amplification using HDA and RPA.

## 1 Introduction

**Fig. 1:**
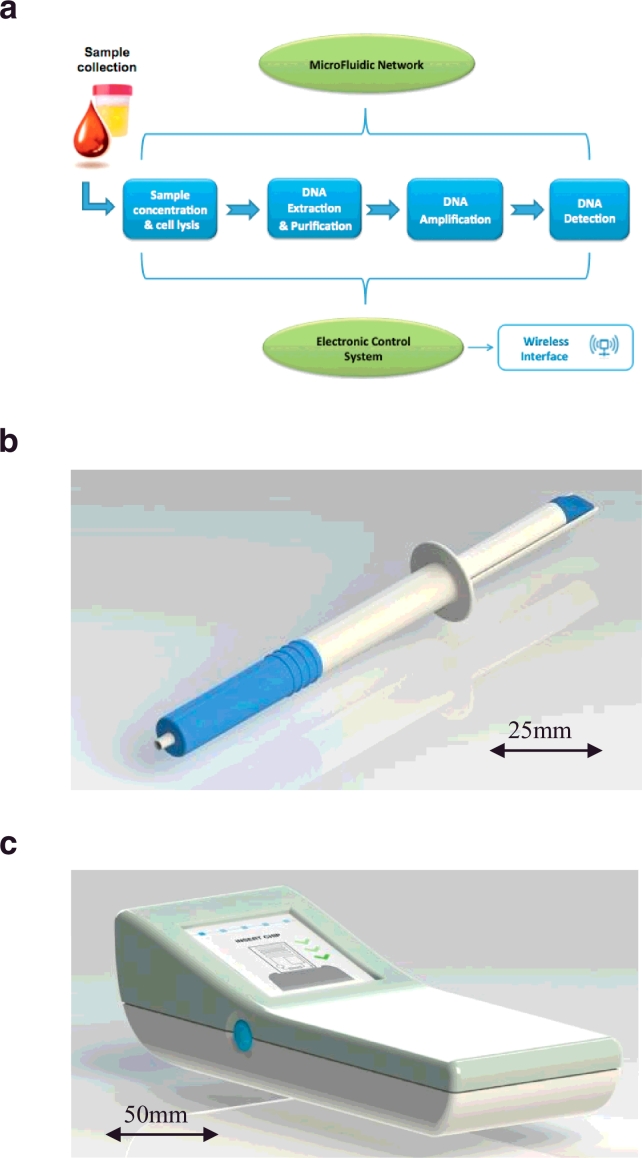
Integrated micro-engineered platform: (a) sample-in-to-answer-out system concept; (b) sample collection device; and (c) envisaged handheld device.

The accurate and rapid identification of pathogens is important in global health to enable immediate and appropriate treatment for vulnerable and hard to reach populations. This is particularly true for sexually transmitted infections (STIs) with the occurrence of extremely drug resistant *Neisseria Gonorrhoea* [1]. Nucleic acid amplification testing (NAAT) has become increasingly used for point of care test (POCT) development due to its potential for high sensitivity and selectivity, but there is a challenge to provide inexpensive, portable and mains-power independent platforms for remote settings that allow for simple sample handling. Sample collection and integration with preparation methods including nucleic acid extraction has inhibited the uptake of commercial POCT devices. A hand-held, battery operated, integrated microengineered platform is under development for sample collection, automated DNA extraction, isothermal amplification [2] and optical detection directly from raw samples such as urine, blood, swabs and saliva (Fig. 1a). Whilst a number of companies have developed point of care devices, for example the Cepheid GeneXpert, Biofire Filmarray and the LIAT analyser, all of the systems are benchtop, require mains power and some hands on sample preparation [3], [4], [5], [6]. The cost of the benchtop devices tend to be high, within this paper the development of a prototype, handheld, low cost amplification and detection platform that cost less than GBP150 for parts and labour is described.

In addition to this, a simple, disposable sample collection device (Fig. 1b) that can be used to collect self-taken urine and swab samples is also being developed. The sample collection device is designed to interface directly with a disposable microfluidic cartridge via a Luer fitting in which the assay is conducted. The sample will be delivered into the cartridge through a plunging mechanism. This device and its corresponding interface mechanism are under development and not described herein. The vision of the project is that the prototype amplification and detection platform described herein will be developed into a handheld device (Fig. 1c) integrating the optics and heating elements along with data acquisition, control and communications hardware. This handheld device will automate sample analysis and send results directly to clinicians, via a mobile phone, for rapid diagnosis to expedite time to treatment. We aimed to initially target genital samples for the rapid diagnosis of STIs.

**Fig. 2:**
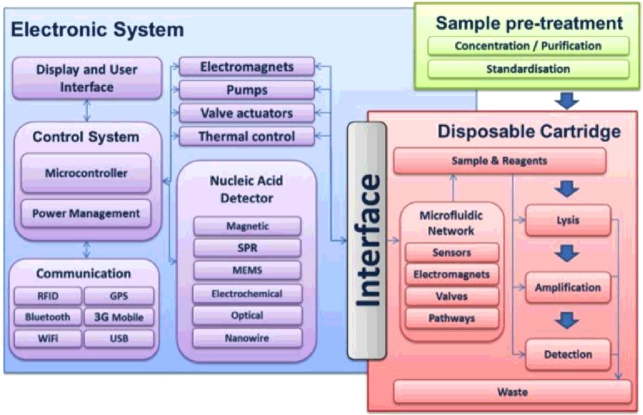
Modular lab on a chip system overview.

The POCT has been developed using a modular process (Fig. 2) enabling any section to be removed and replaced by an alternative method. For example, communications with the handheld device could be achieved using the state-of-the-art wireless technologies or USB depending on the setting in which the device is employed. Similarly sample preparation, isothermal amplification and detection types can be altered depending on the setting and disease type that is being identified.

Nucleic acid extraction for POCT devices is dominated by solid phase extraction with chaotropic salts using silica membrane, columns [7], [8] and magnetic beads [9]; Drawbacks of these methods for POCT development include the required centrifugation for membranes and an external magnetic field for active mixing of magnetic beads, whilst the use of toxic guanidinium thiocyanate can inhibit downstream polymerase chain reaction (PCR) [10]. This paper reports a method of DNA isolation using chitosan impregnated on an organic membrane inserted into polydimethylsiloxane (PDMS)/glass prototyped microfluidic devices. Chitosan was chosen as it simplifies the extraction process and removes the requirement for guanidium thiocyanate. Chitosan is a deacetylated form of chitin. Protonation of amine groups cause chitosan to exist as a polycationic polymer at pH < 6.2, whereas at higher pH, the amine groups are deprotonated. Protonation of the amine groups causes the chitosan to be cationic, thus it adsorbs negatively charged DNA, when deprotonated (at higher pH) the DNA is released into the surrounding solution [11], [12].

Polymerase chain reaction (PCR) was the first method employed for nucleic acid amplification testing (NAAT). More recently isothermal amplification methods have been developed that utilise enzymes for DNA strand separation [1]. Isothermal methods were chosen for this project as they remove the requirement for rapid heating and cooling steps required in PCR, therefore less power is consumed within the handheld device. Optical detection of amplified DNA was chosen as this could be implemented in a low cost manner in a handheld device using off the shelf components, and allows real time visualisation of the NAAT reaction kinetics, the benefit of this over other methods is that the original sample load can be determined [1]. Experiments were conducted using thermophillic Helicase Dependent Amplification (tHDA) and Recombinase Polymerase Amplification (RPA) to show the versatility of the platform.

The following sections describe the fabrication and assembly of the prototype platform, design and optimisation of the heating module and optics. Further, the design of the microfluidic cartridge, on-chip DNA extraction and isothermal amplification are also discussed.

## 2 Platform development

**Fig. 3:**
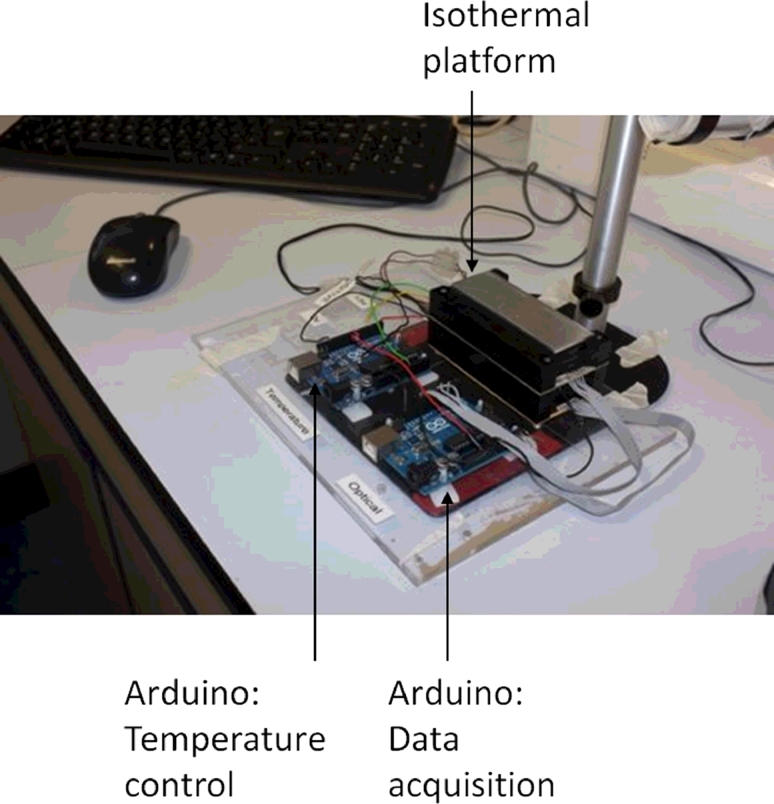
Small footprint, low cost, isothermal amplification platform with two Arduino Uno microcontroller boards for thermal control and data acquisition.

**Fig. 4:**
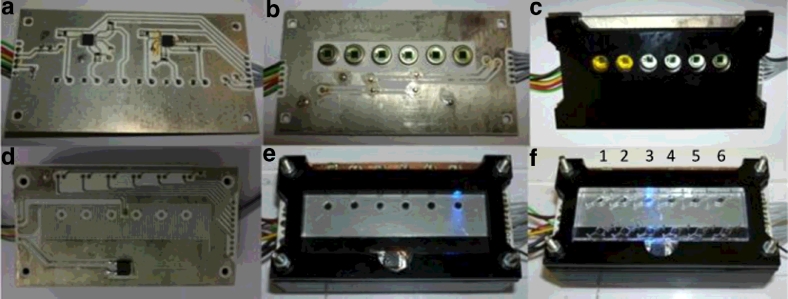
Isothermal amplification platform layers: (a, b) photodiode PCB bottom and top respectively; (c) black laser cut PMMA layer housing six long pass optical filters; (d) resistive heating element PCB with integrated thermistor; (e) the assembled device and aluminium plate for efficient heat distribution across the microfluidic cartridge showing the excitation LED; and (f) the whole platform system with microfluidic cartridge, the LED is exciting chamber 3 within the microfluidic cartridge.

A low cost isothermal amplification and optical detection platform has been developed incorporating a resistive heating element, low cost photodiodes, LEDs and optical filters (Figs. 3 and 4). The control and data acquisition was conducted using two Arduino Uno microcontroller boards.

The platform was produced by assembling the layers of printed circuit boards (PCB) (Fig. 4a–d) with layers of laser cut PMMA as support structures to produce a final device with a height of 32 mm, depth of 54 mm and length of 100 mm. This allows this prototype design to be easily packaged within the envisioned handheld device (Fig. 1c).

### 2.1 Heating element design

A resistive heating element (Fig. 4d) was designed and developed from a two-layer printed circuit board using standard photo-etching methods. A surface mount thermistor was integrated centrally on the heating element to monitor and control temperature changes in the heater. An aluminium plate was attached to this heating element using a heat transfer adhesive as shown in Fig. 4e, to function as an isothermal plate to distribute the heat evenly to the chip and thermistor. The heating element produced sufficient energy to reach the required temperatures; 65 °C to perform the tHDA and 37 °C to perform RPA isothermal NAAT in ≈90 s.

### 2.2 Optics

A low-cost photodiode (BPW21, Centronic, UK) with a high gain operational amplifier (OPA4705, Texas Instruments, USA) with 1 GΩ feedback resistor was utilised to perform the optical detection (Fig. 4a and b). The photodiodes were coupled to the device via a 15 mm long section of hand polished, unjacketed 3000 µm plastic optical fibre (Edmund Optics, Barrington NJ, USA). A long pass filter cut from orange glass (OG515, Schott AG, Germany) was placed between the photodiode and the optical fibre (Fig. 4c). The optical fibre was aligned through a hole on the heating element and on the isothermal plate to rest flush to the upper surface of the isothermal plate where the underside of the microfluidic chambers come to rest. A 3 mm LED (L-7104QBC-D, Kingbright, Taiwan) which illuminated the chip orthogonal to the optical fibre was used to perform the excitation at 470 nm (Fig. 4e).

### 2.3 Control and signal acquisition

The control of the heating element and excitation LEDs, in addition to the acquisition and processing of sensor output, was performed using two Arduino Uno microcontroller boards (Arduino, Turin, Italy). One board was used to control the heating element and the other was used to control the excitation LED and acquire the photosensor data. Control of the heating element was performed using a simple bang-bang control algorithm which triggered in response to thermistor temperature on the heating element. Separate software written in the Arduino environment was used to initiate excitation LEDs and acquire 20 photosensor readings over 300 ms and return an average of readings following a pre-sampling delay to allow for sensor rise time. Fluorescence signal sampling from each chamber was performed in serial order to prevent crosstalk between different reaction chambers. Data output from the microcontroller, time stamp (seconds) and raw fluorescence signal (0–5000 mV), were recorded to study change in fluorescence of the reaction over time. Acquired real-time data was displayed on a PC for analysis and downloaded and further processed to produce Figs. 8–10.

## 3 Methodology

### 3.1 Heating element optimisation

The platform was set to 44 °C and switched on for 10 min to ensure even heat distribution across the isothermal plate. Thermal control was implemented using an Arduino Uno microcontroller board. A 6 well, open test chip was filled with 50 µL of nuclease free water. A digital thermometer and K-type thermocouple were used to measure the temperature in each well, a jig was used to ensure temperature measurements were made at the same height in each fluid filled well.

### 3.2 Microfluidics

The microfluidic cartridge was designed to allow the sample to mix efficiently with the lysis binding buffer prior to the extraction chamber. Due to the laminar nature of the flow within the microfluidic devices (Re < 1), passive mixing was utilised via a serpentine channel. In order to do this, four cartridges of varying design were fabricated and tested experimentally. The cartridges incorporated a 0.5 × 0.5 mm^2^ (width × height) serpentine channel with or without a pre-mixing chamber. The pre-mixing chamber incorporated geometries including a trapezoid, pear and ellipsoid structures.

Microfluidic moulds were designed using Solidworks CAD software and saved as STL files. The moulds were created using 3D printing techniques with an Objet 30 Pro using jetted photopolymer deposition. QSil 218 PDMS (ACC Silicones, UK) was mixed at a ratio of 1:10 for 2 min and then degassed using a centrifugal degasser for 4 min. The moulds were cleaned by rinsing with iso-propanol alcohol and then DI H_2_O to remove contaminants; they were then dried using nitrogen. The mould was placed into a custom made stainless steel frame which was cleaned in the same manner. After degassing the PDMS it is poured gently into the mould and left in the oven at a set temperature of 45 °C for 4 h. The cured PDMS was removed from the mould; a handheld corona treater, BD20-AC (Electro-Technic Products Inc., US), was used to bond the PDMS to a 75×50 mm^2^ glass slide. The nucleic acid extraction membrane was placed in the chamber and the treated surfaces are then pressed together and placed in an oven at 45 °C for 8 h.

**Fig. 5:**
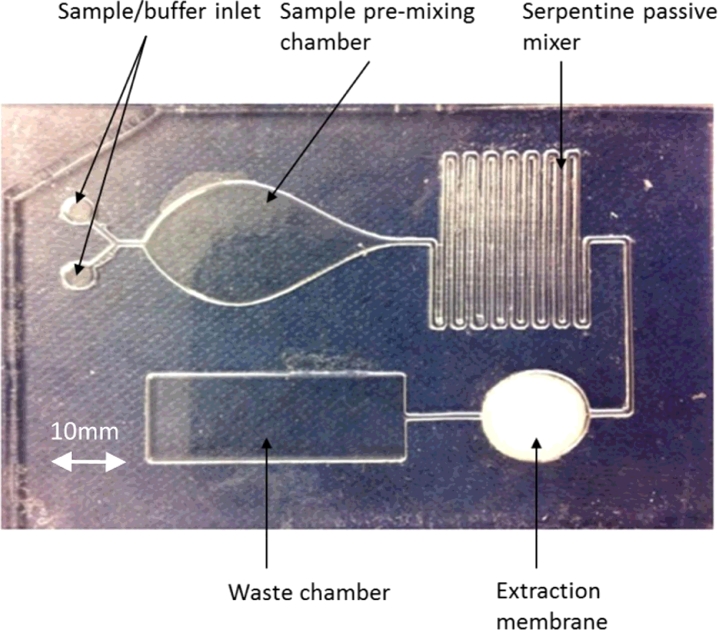
Credit card sized microfluidic extraction cartridge incorporating a passive serpentine mixer.

The microfluidic device incorporates sample and buffer inlets, a pre-mixing chamber, passive serpentine mixer and nucleic acid extraction membrane (Fig. 5).

### 3.3 DNA extraction

Chitosan was dissolved in 2 v/v% acetic acid with varying weight percentages of chitosan (1–5 w/v%). Various grades of Whatman chromatography paper were used as membranes, thus a hybrid plastic paper microfluidic device was created. Membranes were added to the solution and cross-linked using either 1 v/v% glutaraldehyde (GA) or 0.1 v/v% (3-Glycidoxypropyl) methyldiethoxysilane (GPTMS). The membranes were left in solution for eight hours, removed and thoroughly rinsed in 10 mM acetic acid. Membranes were dried in an oven at 60 °C for one hour. The membranes were inserted into a microfluidic chamber with a total volume of 100 μL.

Membranes were tested by spiking TE buffer with salmon sperm DNA with concentrations of 100 ng/μL and 0.1 ng/μL. Further tests were conducted incorporating lysis by purifying DNA from *Escherichia coli* (*E. coli*) spiked phosphate buffered solution using four designs of passive mixing-lysis-extraction cartridges (Fig. 2b). The buffers and sample were loaded into a length of 0.5 mm tubing so that they could be flowed in sequence into the microfluidic chamber. 2-(N-morpholino)ethanesulfonic acid (MES) buffer (10 mM) was prepared at pH 5.0 (pH 3.0 if alkaline lysis was required). Samples were eluted using 10 mM TRIS buffer at pH 9 and pH 9.5. A volume of 25 μL MES buffer was passed over the membrane at a flow rate of 1.53 μL/s, this was left in the chamber for 5 min to ensure protonated amino groups were formed on the chitosan surface. The sample (25 μL) was flowed directly into the chamber with MES buffer solution; this was left for 5 min. The solution was removed from the chamber by flowing air into the device at 3 μL/s. The elution buffer (50 μL) was flowed into the device at 1.53 μL/s and left for 10 min, this was removed and the extraction efficiency was calculated by measuring the concentration of DNA that was eluted from the membrane. This was compared with the original sample, measurements were made using a NanoDrop Spectrophotometer and Qubit HS dsDNA assay with a Qubit Fluorometer. Concurrent experiments were conducted using a Qiagen mini prep commercial DNA extraction kit for comparison.

### 3.4 DNA amplification

Separate microfluidic cartridges containing six individual chambers (Fig. 4f) were designed and manufactured to test isothermal NAAT assays and real time optical detection. NAAT tests were conducted using a one-step tHDA (BioHelix, US) and RPA technologies (TwistDx, UK). The tHDA reactions were performed using the primers and positive control provided with the tHDAIII kit. The master mix was prepared in ratios as described in the test manual [13]. Primers, MGF3 (forward) and NGR3 (reverse) were used at a final concentration of 7.5 nM each and 20 pg control template pCNG1 was added. Reagents were mixed and pipetted manually into the 6 well microfluidic device (Fig. 4f). The microfluidic cartridge was placed onto the pre-heated isothermal plate at 68 °C and data collection was initiated.

An RPA assay (TwistAmp fpg) targeting the *CDS2* gene of *Chlamydia trachomatis* (CT) was designed based on the primer set information provided by [14]. A sequence specific probe for real time optical detection was designed as described in the test manual [15]. The sequences of the primer sets and probe are indicated in Table 1. The RPA reaction mix was made with 2.1 µL of each of the forward and reverse primers and 1.2 µL of the probe with concentrations of 10 µM and 5 µM respectively in an Eppendorf tube containing 29.5 µL of the rehydration buffer. 11.6 µL DNA free water was added to the reaction mix together with 1 µL of CT genomic DNA (VR-348BDۛ *C. trachomatis* genomic DNA BOUR; Serovar E, LGC, UK) containing 1×10^5^ copies to result in a final volume of 47.5 µL. The reaction mix was used to rehydrate the freeze-dried reaction through pipetting and was kept in ice to inhibit reaction initiation. Finally, 2.5 µl of the magnesium salt supplied with the kit was added to the reaction and mixed well through pipetting and manually loaded into the 6 well microfluidic cartridge. The cartridge was then placed onto the pre-heated (10 min) isothermal plate at 44 °C and data collection was initiated.

## 4 Results and discussion

### 4.1 Heating element

**Fig. 6:**
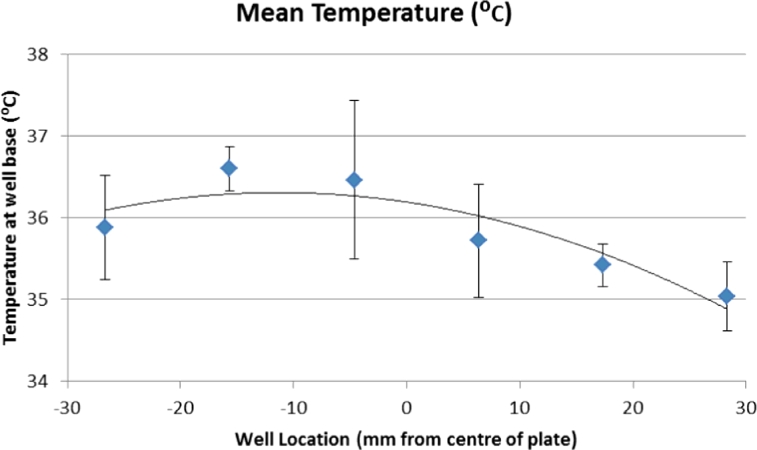
Spatial variation in microfluidic chip reaction chamber temperature.

The resistive heating element provided precise and stable heating of the microfluidic cartridge, a small thermal gradient across the device was seen but this was less than 2 °C. Fig. 6 below indicates the distance of the well from the centre of the heating plate and their corresponding chamber temperatures when the resistive heater temperature was set to 44 °C; temperature measurements were taken five times for each chamber which corresponds to the error bars in Fig. 6. Similar experiments carried out to evaluate the platforms’ capability to provide heating to conduct tHDA amplifications demonstrated that the thermal gradient was smaller than 0.8 °C. The small thermal gradient did not show a variance in the amplification time or gradient when the amplification performance was monitored in the central and outlying chambers.

### 4.2 DNA extraction

**Fig. 7:**
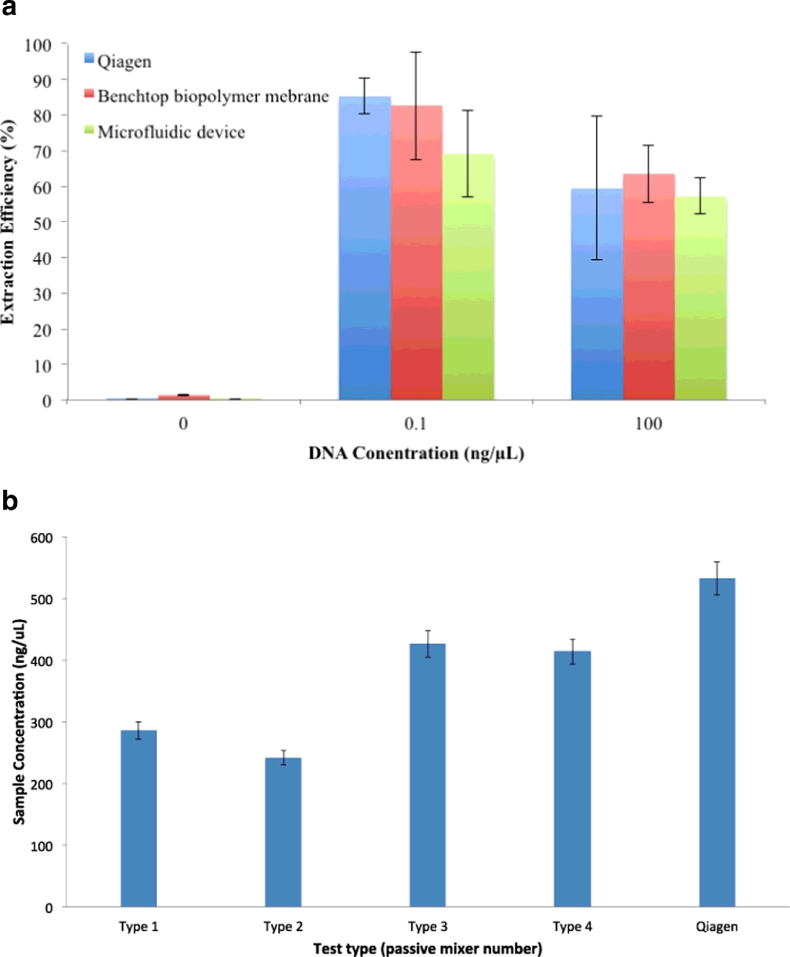
(a) Extraction results for the new membrane compared to a Qiagen spin column extraction with varying concentrations of DNA; and (b) passive mixing device results for DNA extracted from *E. coli* cells

Cross-linking chitosan using GA gave the highest extraction results. However, it was noted that cross-linking was uneven across the surface of the membrane and large variation in the extraction efficiency was seen. The extraction efficiency was lower when high DNA concentrations were used, at 100 ng/μL both the Qiagen and chitosan membranes showed lower extraction efficiencies, the membrane may be saturated and unable to adsorb more DNA (Fig. 7a).

Results for membranes tested on the benchtop, impregnated with 4 w/v% chitosan and cross linked with GA showed extraction efficiencies of 82% and 63% respectively for samples of 0.1 ng/µL and 100 ng/µL. When the membranes were inserted into the microfluidic device the extraction efficiency was lowered to 69% and 57%. These compare to Qiagen spin column results of 85% and 59.4% (Fig. 7a). GPTMS cross linking gave more homogenous results, extraction efficiencies up to 58% have been shown for 0.1 ng/μL using a 1 w/v% chitosan membrane. At higher w/v% chitosan when cross-linked with GPTMS, it was difficult to reverse the protonation of the amino groups and therefore, elute DNA from the membrane at pH 9.0, but when the elution buffer pH was increased to pH 9.5 bound DNA was released. However, efficiency was lowered to <30%. Early experiments showed poor wetting of the chitosan membrane when the MES buffer was introduced, therefore Triton-X 100 was added to the MES protonation buffer, this significantly increased extraction efficiency from 35% to 44.6%. Extraction experiments using *E. coli*, incorporating chemical cell lysis in the passive mixer cartridges showed results with the total extraction efficiency around 80% of the Qiagen kit (Fig. 7b).

### 4.3 DNA amplification

**Fig. 8:**
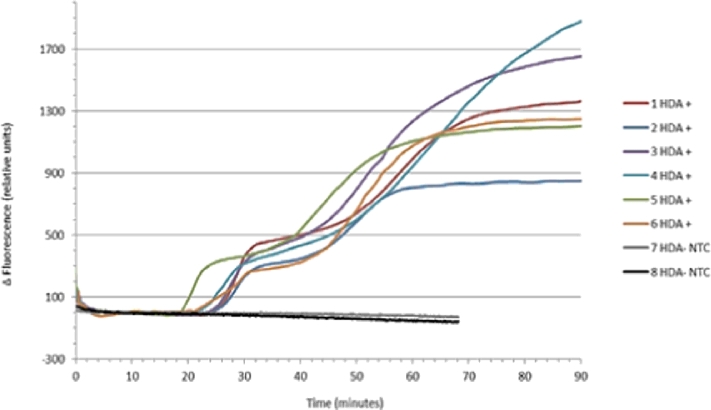
HDA amplification curves from the isothermal amplification platform, positive and negative reactions for 20 pg pCNG.

**Fig. 9:**
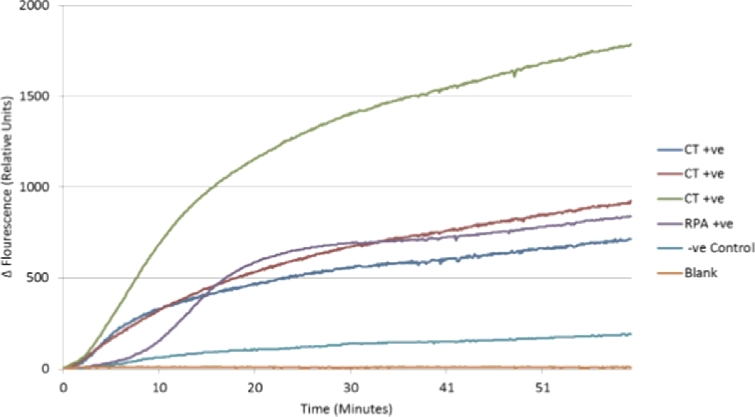
RPA amplification curves of CT genomic DNA. The CT positive wells contained 1×10^5^ copy numbers of CT genomic DNA, RPA positive well contained the positive sample provided with the TwistAmp fpg kit and the negative control well contained all reagents apart from the CT genomic DNA and the blank well contained DNA free water.

**Fig. 10:**
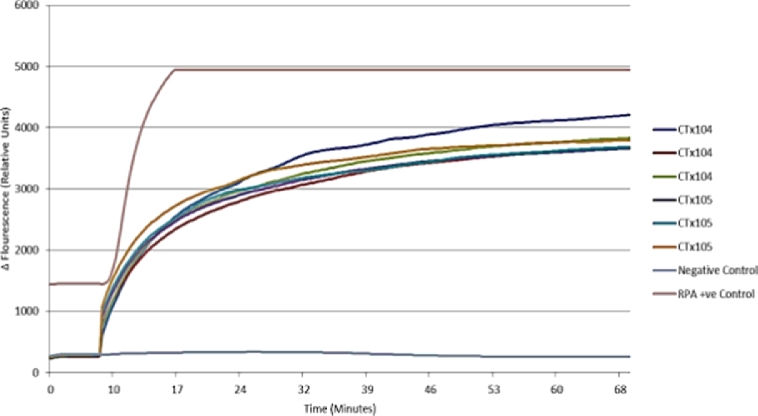
RPA amplification curves from the Axxin T16-ISO platform.

Both isothermal (tHDA and RPA) NAATs performed as expected and were repeatable across the device. The tHDA assay was able to detect 1.32×10^6^ copies of pCNG1 template (Fig. 8) and the RPA assay was able to detect CT genomic DNA within 10 min of the initiation of the reaction (Fig. 9). The preliminary RPA assays contained 1×10^5^ copies of CT genomic DNA and currently further studies are being carried out to evaluate the platforms’ performance with low copy numbers of DNA. Assays run on the developed amplification and detection platform were run concurrently on an Axxin T16-ISO platform that showed similar reaction kinetics for both tHDA and RPA assays, this is shown in Fig. 10 using RPA negative control, RPA positive control, 1×10^6^, 1×10^5^ copies and of CT DNA.

The results show that the prototype platform can emerge as a platform for iNAAT diagnostic assays. Additionally, the modular nature of this platform enables it to be utilised as an assay development tool or a diagnostic tool. Provided the low cost to fabricate the platform, and its suitability to be packaged within a handheld standalone unit, it is anticipated that this platform will produce a true point-of-care diagnostic device for molecular diagnostics. The limit of detection of the platform at present is substantially high; however, this can be considerably reduced by optimising the DNA extraction efficiency and amplification assay. Further, based on the results, it is evident that a detectable signal can be produced from a raw sample within 40 min of reaction initiation.

## 5 Conclusions

The work presented here shows a low cost, rapid nucleic acid extraction, isothermal amplification and detection platform. Nucleic acid extraction showed similar results to the commercial extraction kits, work is on-going to further optimise the results using clinical samples. This nucleic acid extraction method does present an advantageous solution, extraction time is reduced to less than 20 min and is extremely low cost, and this can also be an advantageous method for those working in the paper microfluidic field. The isothermal amplification platform and resistive heating element proved a robust method to amplify nucleic acid using isothermal assays and has shown repeatable results. The simple optics setup demonstrated high sensitivity and quick detection of the tHDA and RPA reactions eliminating the requirement for expensive dichroic filters and lenses. The group is now developing a new microfluidic cartridge with integrated nucleic acid extraction, which fits into the current low cost isothermal amplification platform. This will have the ability to prove the sample-in-to-answer-out capability of the platform. The platform will be further developed to create a robust handheld device with disposable microfluidic cartridges and simple, easy to use, sample collection at much lower cost than comparable systems creating a versatile portable test for STIs.

## Conflicts of interest

The authors declare no conflict of interest.

## Figures and Tables

**Table 1:** *Chlamydia trachomatis* specific amplification primers and probe sequences used within the RPA isothermal amplification assays.

Primer/probe name	Size (bp)	Sequence (5ʹ–3ʹ)
CDS2-FW (forward primer)	33	CCT TCA TTA TGT CGG AGT CTG AGC ACC CTA GGC
CDS2-RV (reverse primer)	32	CTC TCA AGC AGG ACT ACA AGC TGC AAT CCC TT
fpg Modified probe	33	[5ʹ BHQ1] GTT T[dR-FAM] T ACT CCG TCA CAG CGG TTG CTC GAA GCA [3ʹ-block]
